# The impact of the COVID-19 pandemic on 397 631 elective dental admissions among the under-25s in England: a retrospective study

**DOI:** 10.1093/pubmed/fdae058

**Published:** 2024-05-03

**Authors:** Puji Faitna, Dougal S Hargreaves, Francesca K Neale, Simon E Kenny, Russell M Viner, Paul P Aylin, Alex Bottle, Paul Ashley

**Affiliations:** Department of Primary Care and Public Health, School of Public Health, Imperial College London, London, W6 8RP, UK; Department of Primary Care and Public Health, School of Public Health, Imperial College London, London, W6 8RP, UK; Mohn Centre for Children’s Health and Wellbeing, Imperial College London, London, W6 8RP, UK; Department of Primary Care and Public Health, School of Public Health, Imperial College London, London, W6 8RP, UK; Department of Paediatric Surgery, Alder Hey Children’s Hospital, Liverpool, L14 5AB, UK; NHS England and NHS Improvement, London, SE1 8UG, UK; Institute of Systems, Molecular and Integrative Biology, University of Liverpool, Liverpool, L69 7BE, UK; Population, Policy and Practice Research Programme, UCL Institute Great Ormond Street Institute of Child Health Population Policy and Practice, London, WC1N 1EH, UK; Department of Primary Care and Public Health, School of Public Health, Imperial College London, London, W6 8RP, UK; Department of Primary Care and Public Health, School of Public Health, Imperial College London, London, W6 8RP, UK; Eastman Dental Institute, University College London, London, WC1E 6DE, UK; School of Life and Medical Sciences, University College London, London, W1T 7NF, UK

**Keywords:** child, adolescent, COVID-19, pandemics, tooth extraction, hospitals, dental surgery

## Abstract

**Background:**

COVID-19 caused widespread disruptions to health services worldwide, including reductions in elective surgery. Tooth extractions are among the most common reasons for elective surgery among children and young people (CYP). It is unclear how COVID-19 affected elective dental surgeries in hospitals over multiple pandemic waves at a national level.

**Methods:**

Elective dental tooth extraction admissions were selected using Hospital Episode Statistics. Admission trends for the first 14 pandemic months were compared with the previous five years and results were stratified by age (under-11s, 11–16s, 17–24s).

**Results:**

The most socioeconomically deprived CYP comprised the largest proportion of elective dental tooth extraction admissions. In April 2020, admissions dropped by >95%. In absolute terms, the biggest reduction was in April (11–16s: −1339 admissions, 95% CI −1411 to −1267; 17–24s: −1600, −1678 to −1521) and May 2020 (under-11s: −2857, −2962 to −2752). Admissions differed by socioeconomic deprivation for the under-11s (*P* < 0.0001), driven by fewer admissions than expected by the most deprived and more by the most affluent during the pandemic.

**Conclusion:**

Elective tooth extractions dropped most in April 2020, remaining below pre-pandemic levels throughout the study. Despite being the most likely to be admitted, the most deprived under-11s had the largest reductions in admissions relative to other groups.

## Introduction

The COVID-19 pandemic caused widespread disruption to health services worldwide. In order to treat COVID-19 patients, lower-risk patients (including children and young people (CYP)) were de-prioritized. Globally, there were widespread reductions in elective surgery, with an estimated 28 million operations cancelled in the 12 weeks of peak disruption in 2020 alone.^1^

In the UK, strict lockdown measures led to changes in health-seeking behaviour, such as reductions in emergency department attendance,^2^ hospital admissions,^3^ general practitioner consultations^4^ and outpatient appointments.^5^ To create capacity for COVID-19 patients, NHS England advised hospital trusts to reduce planned elective surgeries, and the Chief Dental Officer recommended that routine, non-urgent dental care be deferred where possible.^6^^,^^7^

Delayed elective surgery can have long-lasting impacts on CYPs’ social and emotional development and their families.^8^^,^^9^ Prolonged pain from postponed dental treatment can impact sleep and school attendance, in addition to parents and carers often needing to miss days from work.^10^ By December 2020, NHS England reported that 210 000 children were awaiting elective surgeries and that 66 000 had been on the waiting list for over six months,^11^ raising important concerns about what disruptions may have also occurred within general dental practice and the impacts of both these delays on CYP.^12^

No published studies to date have quantified what impact COVID-19 has had on dental elective surgeries in England across the three pandemic waves and compared them with five pre-pandemic years.

### Aims

Primary aim: to describe month-by-month and overall trends in elective dental admissions during the pandemic and compare these with the preceding five years.

Secondary aim: to investigate admission trends by patient age, deprivation and ethnicity.

## Methods

### Data sources and cohort definition

England’s hospital episode statistics (HES) admitted patient care data were provided by NHS Digital. HES is a hospital administrative database on all NHS hospitals and private hospital admissions paid for directly by the NHS and contains information on patient characteristics and procedures conducted. Dental extractions performed under general anaesthesia by community dental services that subcontract hospitals may not be fully captured within HES. Procedures were coded using the UK’s OPCS clinical coding convention, and individuals with at least one hospital spell were included between 1 Feb 2015 and 31 Mar 2021. Data access beyond 31 Mar 2021 was unavailable at the time of writing. Area-level socio-economic deprivation in HES is measured using the Index of Multiple Deprivation.^13^

All elective admissions with the main operating procedure as either F09^14–17^ ‘Surgical removal of the tooth’ or F10^14^^,^^15^ ‘Simple extraction of the tooth’ by individuals aged <25 years at the time of admission were selected. Results were stratified into the age groups: 0 to 11 years (under-11s), 11 to 16 years (11–16s) and 17 to 24s (17–24s) to reflect different conditions broadly. The under-11s admitted with these procedures as their primary condition were assumed to mostly reflect dental caries or tooth decay. The 11–16s and 17–24s were more likely to represent impacted teeth (including wisdom teeth) and other orthodontic procedures.

### Exposure and outcomes

The pre-pandemic period was defined as 1 Feb 2015 to 31 Jan 2020, and the pandemic period as 1 Feb 2020 to 31 Mar 2021, covering the first three national lockdowns roughly defined as late March to June 2020, November 2020 and January to March 2021.^18^ The study’s pandemic period included February 2020, as the first cases of COVID-19 diagnosed in England occurred in late January 2020.^19^

The primary outcomes were monthly elective dental admission trends during the pandemic compared with the pre-pandemic period. The secondary outcomes were to report and compare admission trends by deprivation quintile and ethnicity by pandemic period.

### Statistical analyses

Three-monthly pre-pandemic trends were plotted. The pre-pandemic monthly mean was plotted, where each month reflected the monthly mean for the 5 pre-pandemic years. This plot was then joined to the pandemic monthly count. The difference (diff) between the observed (O) and expected (E) monthly figures were reported based on the pre-pandemic mean and then summed over the pandemic to report the total reduction in admissions over time to avoid seasonal biases. The percentage drop was calculated by dividing the difference by the expected for each pandemic month, and the range was reported.

Pearson’s Chi-squared test (or Fisher’s exact test) with standardized residuals was applied to test differences between the pandemic periods. The interaction between deprivation and ethnicity was reported using a ratio of admissions. At the time of analysis, the most recent (2021) census data detailing population proportions by ethnicity were unavailable. A comparison of admission counts between pandemic periods was applied to mitigate this.

All analyses used SAS software V.9.4; the statistically significant threshold was 0.05.

## Results

Table 1 describes the patient characteristics of elective dental admissions for the entire study period.

**Table 1:** Patient characteristics by pandemic period

Feature	Value	Period	
		Pre-pandemic (Feb 2015–Jan 2020) *N* = 363,856 (%)	Pandemic (Feb 2020–Mar 2021) *N* = 33,775 (%)
Age	Mean (SD) 0–10 11–16 17–24	12.0 (6.6) 180 926 (49.7) 84 611 (23.3) 98 319 (27.0)	11.6 (6.3) 17 837 (52.8) 8141 (24.1) 7797 (23.1)
Gender	Male Female	168 913 (46.4) 194 943 (53.6)	16 153 (47.8) 17 622 (52.2)
Deprivation quintile	1 (least deprived) 2 3 4 5 (most deprived) 6 (unknown)	49 469 (13.6) 54 433 (15.0) 62 170 (17.1) 79 895 (22.0) 115 469 (31.7) 2420 (0.7)	4782 (14.2) 5220 (15.5) 5703 (16.9) 7284 (21.6) 10 630 (31.5) 156 (0.5)
Ethnic group	Black or Black British Asian or Asian British White Other (incl. mixed) Unknown	11 967 (3.3) 27 264 (7.5) 228 082 (62.7) 21 162 (5.8) 75 381 (20.7)	1129 (3.3) 2493 (7.4) 20 068 (59.4) 2127 (6.3) 7958 (23.6)
Admission source	Home Transfer from acute hospital Transfer from other hospital Other/unknown	357 883 (98.4) 5 (<0.1) 4952 (1.4) 1016 (0.3)	33 061 (97.9) *Suppressed 636 (1.9) 77 (0.2)
Emergency admissions in previous 12 months	0 1 2 3+	342 982 (94.3) 16 324 (4.5) 2842 (0.8) 1708 (0.5)	31 768 (94.1) 1500 (4.4) 294 (0.9) 213 (0.6)

^*^Suppressed due to small numbers.

### Admissions

**Fig. 1:**
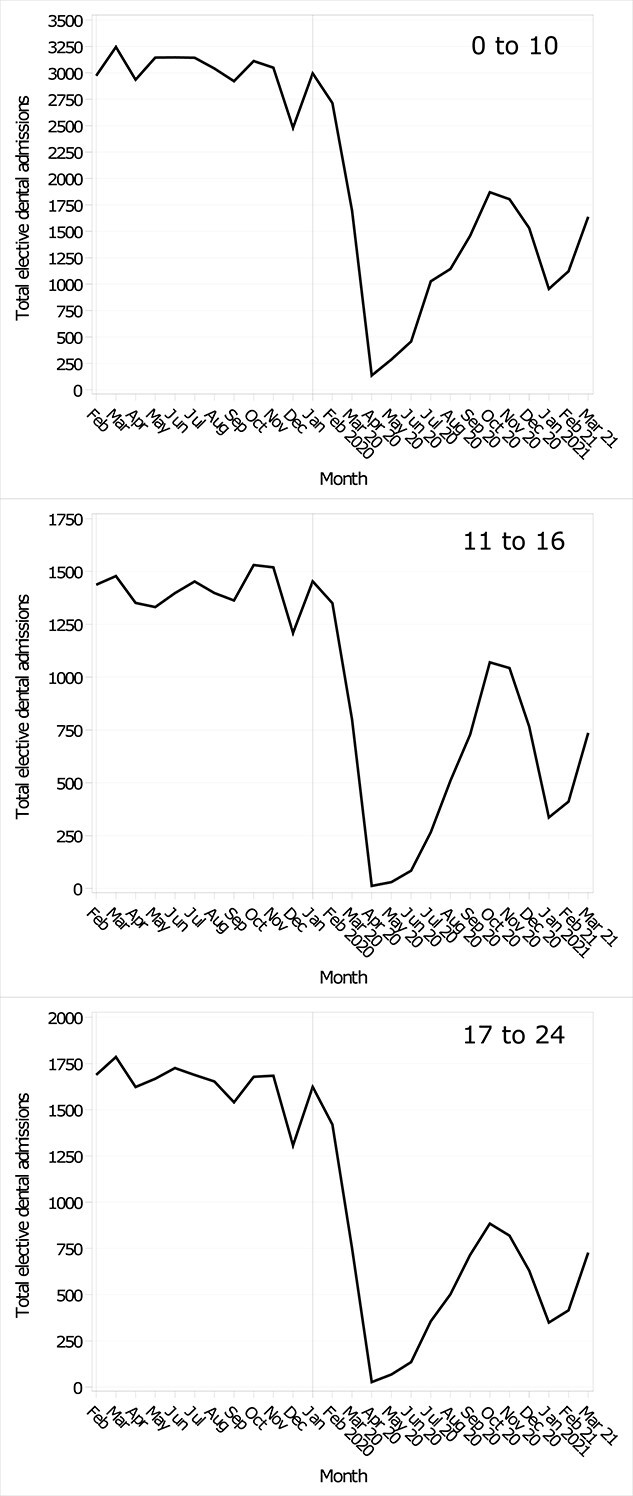
Monthly elective dental admission trends during the pandemic and the mean monthly admissions for the previous five years, stratified by age group.

Before the pandemic, elective dental admissions had a steady downward trend (Fig. S1). There was a marked statistically significant reduction in admissions for all pandemic months when compared with the pre-pandemic average (Fig. 1), most notably in April 2020, where admissions fell by 95.4% for the under-11s, 99.1% for the 11–16s and 98.3% for the 17–24s (Table S1). After April 2020, the percentage fall ranged from −90.9% to −38.3% for the under-11s, −97.7% to −30.1% for the 11–16s and − 95.9% to −47.3% for the 17–24s (Table S1).

In absolute terms, the steepest reduction in admissions was in April 2020 for the 11–16s (−1339 admissions (95% CI -1411 to −1267) and 17–24s (−1600 (−1678 to −1521) and May 2020 for the under-11s (−2857 admissions (95% CI -2962 to −2752) (Table, S1). A recovery in admissions followed until October 2020, when admissions dipped until January 2021, when admissions started to recover again. Although there was some recovery, admissions had not returned to pre-pandemic levels by March 2021.

The total reduction in admissions amounted to 24 567 fewer admissions for the under-11s, 11 697 fewer admissions for the 11–16s and 15 340 fewer for the 17–24s. Overall, the pandemic had 51 604 fewer elective dental admissions than expected compared with the previous five years.

### Admissions by socioeconomic deprivation quintile

**Fig. 2:**
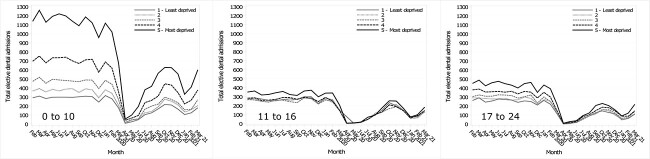
Monthly elective dental admission trends by socioeconomic deprivation quintile during the pandemic and the mean monthly admissions for the previous five years, stratified by age group.

Before the pandemic, when admissions were analysed by socioeconomic deprivation quintile, the most deprived CYP had the most admissions (Fig. 2). Similar results were found for the pre-pandemic plots, as the median socioeconomic deprivation quintiles for all admissions were consistently the second most deprived quintile (Fig. S2). The pattern of reduction and recovery in admissions seen in the whole sample was replicated when analysed by socioeconomicdeprivation quintiles (Fig. S3–5).

The percentage drop in admissions was steepest in April 2020 for all age groups, the differences ranging from 94.0% to 97.2% for the under-11s, more than 98.0% for the 11–16s and 97.9% to over 98.0% for the 17–24s across the socioeconomic deprivation quintiles. The percentage changes after April 2020 ranged from 26.9% to 91.6% for the under-11s, 21.9% to over 98.0% for the 11–16s and 43.1% to 98.0% for the 17–24s (Table S3–S5).

In absolute terms, the sum of the admission reductions for the pandemic period by socioeconomic deprivation quintile was largest among the most deprived CYP for the under-11s (1: Least deprived −2207 admissions; 2: −2852; 3: −3887; 4: −5765; 5: Most deprived −9604), 11–16s (1: Least deprived −2214; 2: −2128; 3: −2110; 4: −2355; 5: Most deprived −2830) and 17–24s (1: Least deprived −2388, 2: −2563; 3: −2880; 4: −3353 and 5: Most deprived −4081) (TableS3–S5).

When comparing socioeconomic deprivation by pre-pandemic and pandemic periods overall, rather than month-by-month, the reductions in elective dental admissions for CYP aged under-11 during the pandemic were statistically significant (*P* < 0.0001), while the 11–16s (*P* = 0.601) and 17–24s (*P* = 0.342) were not statistically significant. Standardized residuals for the under-11s show more than expected admissions for the least and second least deprived socioeconomic quintiles during the pandemic (Table S6) and fewer than expected admissions by CYP from the most socioeconomically deprived quintile. This was not seen in 11- to 24-year-olds.

### Admissions by ethnicity

**Fig. 3:**
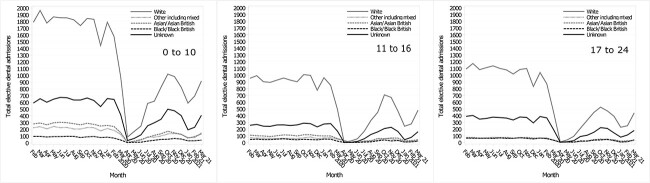
Monthly elective dental admission trends by ethnicity during the pandemic and the mean monthly admissions for the previous five years, stratified by age group.

Before the pandemic, elective dental admissions were most frequently made by CYP coded as ‘White’, followed by ‘Unknown’ (Fig. 3). CYP coded as ‘Unknown’ ethnicity in the pre-pandemic period was 20.7%, increasing slightly to 23.6% during the pandemic period. The pattern of reduction and recovery in admissions seen in the whole sample was replicated when analysed by ethnicity (Fig. 3).

Changes over time by ethnicity differed by age (*P* < 0.0001). For the under-11s, the significance was driven most by more than expected admissions of CYP coded as ‘Unknown’ ethnicity during the pandemic (Table S7). The significance for the under-11s was also driven by fewer than expected admissions for ethnicities ‘White’ and ‘Asian/Asian British’ during the pandemic and fewer than expected admissions for the ‘Unknown’ ethnicity in the pre-pandemic period (Table S7).

Like the under-11s, the significant difference between pandemic periods for the 11–16s was driven most by more than expected elective dental admissions for the ‘Unknown’ ethnicity group during the pandemic (Table S8). More than expected admissions by CYP coded as ‘Other including mixed’ during the pandemic period and fewer than expected admissions by CYP coded as ‘White’ during the pandemic also drove the significant difference (Table S8).

For the 17–24s, the largest driver of the significance was the fewer than expected elective dental admissions among CYP coded as ‘White’ ethnicity during the pandemic period. There were also significantly more than expected elective admissions among CYP coded as Asian/Asian British, Unknown and Black/Black British (Table S9).

### Interaction between socioeconomic deprivation and ethnicity on admissions

Overall, each ethnicity category had a significant deprivation gradient (Table S10). The more deprived the CYP, the higher the likelihood of being admitted to the hospital for an elective dental procedure, with variation in slope and shape (Table S10). In absolute terms, the admission counts were largest among CYP coded as ‘White’ and ‘Unknown’. There were fewer elective admissions among the other ethnicity subcategories, making the estimates with more admissions the most robust. For the under-11s, the largest relative difference in admissions between the most and least deprived CYP was among the Asian/Asian British CYP, which saw a 20-fold difference in admissions, followed by Black/Black British CYP, which saw a 9.3-fold difference. For the older age groups, the largest relative difference was among CYP coded as Black/Black British (11–16s: 14.0-fold; 17–24s: 21.0-fold) followed by Asian/Asian British (11–16s: 6.5-fold; 17–24s: 10.5-fold) (Table S10).

## Discussion

### Main findings of this study

Before the pandemic, the most socioeconomically deprived CYP had the greatest number of elective dental admissions for tooth extractions. There was a major decrease in admissions in April 2020, followed by some recovery until October 2020, when admissions dropped again until January 2021. The percentage reduction in April 2020 ranged from 95.4% to 99.1% across age groups. The percentage drop after April 2020 ranged from 21.9% to more than 98.0% across age groups. The under-11s had the largest absolute reduction in admissions. Admission trends were slow to recover and did not return to pre-pandemic levels throughout the 14 pandemic months. Among the under-11s (who comprised about half the admissions), there were significant changes in admissions by socio-economic deprivation quintile, driven by more than expected admissions by the two most affluent deprivation quintiles and fewer than expected admissions by the most socioeconomically deprived CYP during the pandemic. When admissions by ethnicity were compared across pandemic periods, there were statistically significant reductions but mixed findings around which ethnicity groups were driving this finding by age.

### What is already known on this topic

One of the most common reasons for children to be admitted to hospital for elective surgery is tooth extractions.^9^ In the UK, dental caries is a long-standing, preventable public health problem for CYP^20^ that is more prevalent in deprived groups and ethnic minorities.^21^^,^^22^ CYP in England have higher dental health inequalities among children than in Wales and northern Ireland, despite lower average disease levels.^23^

Our findings were broadly consistent with the international^24–27^ and national^28–30^ literature. The pandemic drastically reduced emergency dental procedures in Switzerland^24^ and China.^25^ Similarly, paediatric dental procedures dropped in Brazil^27^ and the US,^26^ with significant variations by region. In one US study, paediatric dental procedures rebounded by August 2020 among the privately insured but remained below pre-pandemic levels among those publicly insured.^26^

To our knowledge, the most recent published study reporting the impact of the pandemic on elective dental admissions among English CYP reports up to August 2020.^29^ The same study reported similar reductions in dental-caries-related tooth extractions for April 2020, and found that the most socioeconomically deprived CYP had the largest reductions in elective dental admissions when compared with the most socio-economically affluent CYP.^29^

### What this study adds

This study quantified the impact of the first 14 pandemic months on hospital admissions for elective dental procedures on a national level among the under-25s. The study’s findings provide context for decision-makers to address the backlog of elective surgeries for CYP, gain a deeper understanding of how pre-existing health inequalities may have impacted CYP’s access to elective dental procedures during the pandemic, and learn from and pro-actively plan for possible future pandemics. This study highlights a concerning finding that dental elective surgery services performed in hospitals did not return to pre-pandemic levels in the 14 pandemic months, and one possible interpretation is that it reflects an ongoing lack of urgency in the recovery of dental services.

It is unclear why there has been a steady downward trend in admissions by the under-25s before the pandemic and if this trend differed depending on the CYP’s socio-economic position or ethnicity. The pre-pandemic admission trend could be influenced by, but not limited to, an overall reduced capacity, reduced access to dental surgeries, the introduction of taxes on soft drinks^31^ or the lowered recognition of dental problems because of reduced access. These possible reasons for the pre-pandemic admission trend are not within the scope of this study but could serve as an important focus for future research. The continuation of this pre-existing decline in admissions could be influenced by a complex range of system pressures, such as funding constraints, workforce disputes and the recent industrial action.

The pre-existing inequality among the under-11s, whereby preventable procedures for dental caries occurred predominantly amongst the most socioeconomically deprived CYP, is further intensified by a slower recovery in admissions than when compared with the most affluent CYP, highlighting the need for appropriate investment into addressing these inequalities. Children from more socioeconomically deprived backgrounds, who already face challenges such as experiencing higher rates of disruptions to their learning and educational outcomes and food insecurity, must not be further disadvantaged by the impacts tooth pain can have on CYP, such as worsened educational outcomes, increased anxiety, which can have further negative impacts on oral health and eating habits. These findings also highlight the importance of ensuring that elective dental procedures, which were often deemed as less urgent during the height of the pandemic, are appropriately prioritized and planned for in possible future pandemics. As well as ensuring that elective DGA is not de-prioritized, it may also be appropriate to consider other strategies, such as prioritizing patients for dental surgery who are already experiencing other inequalities. For example, the British Society of Paediatric Dentistry has issued a blueprint to improve children’s oral health in the UK.^32^ Finally, and most importantly, they are a reminder to decision-makers that this situation could be avoided if this preventable condition was managed in a more upstream way.

This study focused on elective dental procedures among CYP, but future studies could investigate if there was a parallel impact on emergency dental procedures. Future research could also examine the pathways of why this preventable public health problem continues to greatly impact the most socioeconomically deprived and examine what impacts these delays may have had on CYP, their education, mental health and families. More needs to be done to improve the recording of DGA provision across England within HES to facilitate service equity. Future research directions could investigate why there was a delay in admissions returning to pre-pandemic levels and how the current context of ongoing funding and workforce pressures, reduced primary care access and increased demands on hospital capacity may have played a role, which could be used to inform health service planning.

### Limitations to the study

Despite procedure codes having more than 95% accuracy within HES,^33^ there are well-established ethnicity coding issues.^34–36^ Ethnicity was coded ‘Unknown’ for more than one in five admissions in our study and increased from 20.7% to 23.6% in the pandemic, highlighting the possible overrepresentation of non-white ethnicities coded as ‘Unknown’ and possibly distorting the true impact of ethnic disparities on elective dental admissions over the pandemic. At the time of analysis, the most up-to-date national proportions of ethnicity across the country were unavailable. To mitigate the possibility that many ethnic minorities were coded as ‘Unknown’, ethnicity proportions were compared between the pre-pandemic and pandemic periods instead. Another limitation is that it does not link to social or educational data, which could provide insights into how these changes to dental elective surgeries may have impacted children and their families. Many parents and carers were asked to stay home during the pandemic, possibly meaning fewer requests for time off work to care for children with dental pain. However, parental stress was reported to be higher during the pandemic, than when compared with before the pandemic,^37^^,^^38^ indicating families were under more stress than usual, some more than others.^38^

It is difficult to draw causal relationships from these findings due to the retrospective cohort study design and other possible unmeasured confounders such as geographical variations on access to elective dental services. Moreover, HES will not fully capture all dental general anaesthesia (DGA) procedures provided by external organizations using hospital facilities, such as community dental services.^39^ At least 25% of dental DGA providers do not appear on the HES database at all and it is unlikely that all the activity by those DGA providers on the database will be captured accurately.^39^ This study’s dataset does not provide information on general dental practices, so is unable to provide insights on how the pandemic may have directly impacted general dental practices.

Strengths of this study include the ability of HES to capture a large national sample size to allow for month-by-month and overall period comparisons over multiple pandemic periods. Additionally, analysing five years of pre-pandemic admissions allows for a more precise understanding of the pre-pandemic context when interpreting our findings.

## Supplementary Material

Appendix_elective_paper_V7_final_fdae058

## Data Availability

The pseudonymized patient data that were used for this study can be accessed by contacting NHS Digital (see https://digital.nhs.uk/services/data-access-request-service-dars). Access to these data is subject to a data sharing agreement containing detailed terms and conditions of use following protocol approval from NHS Digital. Documents such as the study protocol are not available.

## Notes

**Puji Faitna**, PhD student in Public Health

**Dougal S. Hargreaves**, Houston Reader in Paediatrics and Population Health and Paediatrician

**Francesca K. Neale**, Medical Doctor

**Simon E. Kenny**, National Clinical Director for Children and Young People for NHS England and Consultant Paediatric Surgeon

**Russell M. Viner**, Chief Scientific Advisor to the Department of Education and Professor in Adolescent Health

**Paul P. Aylin**, Professor of Epidemiology and Public Health

**Alex Bottle**, Professor of Medical Statistics

**Paul Ashley**, Professor of Paediatric Dentistry
