# Supplementary material for: The impact of the COVID-19 pandemic on 397 631 elective dental admissions among the under-25s in England: a retrospective study
Source: J Public Health (Oxf). 2024 May 3;46(3):e380–8. doi: 10.1093/pubmed/fdae058 (PMC11358625; doi:10.1093/pubmed/fdae058)
Supplement: Appendix_elective_paper_V7_final_fdae058 [file appendix_elective_paper_v7_final_fdae058.docx]

# Appendix

Table of Contents

[Appendix 1](#_Toc163566967)

[Figure S1. Pre-pandemic total dental elective admissions trend 1](#_Toc163566968)

[Table S1: The difference between monthly elective dental admissions during the pandemic and the five-year pre-pandemic monthly mean by age group. 2](#_Toc163566969)

[Figure S2. Pre-pandemic median deprivation quintile by quarter 3](#_Toc163566970)

[Table S3: The difference between monthly elective dental admissions during the pandemic and the five-year pre-pandemic monthly mean by deprivation quintile for the 0-10s 3](#_Toc163566971)

[Table S4: The difference between monthly elective dental admissions during the pandemic and the five-year pre-pandemic monthly mean by deprivation quintile for the 11-16s 4](#_Toc163566972)

[Table S5: The difference between monthly elective dental admissions during the pandemic and the five-year pre-pandemic monthly mean by deprivation quintile for the 17-24s 5](#_Toc163566973)

[Table S6: Standard residuals for the chi-squared test between pandemic periods by deprivation quintiles for the 0-10s 6](#_Toc163566974)

[Table S7: Standard residuals for the chi-squared test between pandemic periods by ethnicity for the 0-10s 6](#_Toc163566975)

[Table S8: Standard residuals for the chi-squared test between pandemic periods by ethnicity for the 11-16s 6](#_Toc163566976)

[Table S9: Standard residuals for the chi-squared test between pandemic periods by ethnicity for the 17-24s 7](#_Toc163566977)

[Table S10: Count ratios of admissions after modelling the interaction between deprivation quintile and ethnicity by pandemic period and age group. 7](#_Toc163566978)

### Figure S1. Pre-pandemic total dental elective admissions trend

### Table S1: The difference between monthly elective dental admissions during the pandemic and the five-year pre-pandemic monthly mean by age group.

|  | 0 to 10 years | | | | 11 to 16 years | | | | 17 to 24 years | | | |
| --- | --- | --- | --- | --- | --- | --- | --- | --- | --- | --- | --- | --- |
| Month, year | O | E | Diff (95% CI) | % Diff | O | E | Diff (95% CI) | % Diff | O | E | Diff (95% CI) | % Diff |
| Feb, 2020 | 2714 | 2973.6 | -259.6 (-291.2 to -228.0) | -8.7 | 1350 | 1437.4 | -87.4 (-105.7 to -69.1) | -6.1 | 1420 | 1688.8 | -268.8 (-300.9 to -236.7) | -15.9 |
| Mar | 1695 | 3244.4 | -1549.4 (-1626.6 to -1472.0) | -47.8 | 799 | 1478.2 | -679.2 (-730.3 to -628.1) | -45.9 | 750 | 1785.2 | -1035.2 (-1098.3 to -972.0) | -58.0 |
| Apr | 136 | 2935.0 | -2799.0 (-2902.7 to -2695.3) | -95.4 | 12 | 1351.2 | -1339.2 (-1410.9 to -1267.0) | -99.1 | 27 | 1622.8 | -1595.8 (-1674.1 to -1517 | -98.3 |
| May | 287 | 3144.4 | -2857.4 (-2962.2 to -2752 | -90.9 | 30 | 1331.8 | -1301.8 (-1372.5 to -1231 | -97.7 | 68 | 1667.6 | -1599.6 (-1678 to -1521.2 | -95.9 |
| Jun | 457 | 3146.2 | -2689.2 (-2790.8 to -2587 | -85.5 | 84 | 1397.6 | -1313.6 (-1384.6 to -1242 | -94.0 | 135 | 1725.4 | -1590.4 (-1668.6 to -1512 | -92.2 |
| Jul | 1027 | 3143.0 | -2116 (-2206.2 to -2025.8 | -67.3 | 266 | 1452.8 | -1186.8 (-1254.3 to -1119 | -81.7 | 356 | 1688.0 | -1332 (-1403.5 to -1260.5 | -78.9 |
| Aug | 1144 | 3041.4 | -1897.4 (-1982.8 to -1812 | -62.4 | 510 | 1398.2 | -888.2 (-946.6 to -829.8) | -63.5 | 502 | 1652.8 | -1150.8 (-1217.3 to -1084 | -69.6 |
| Sep | 1458 | 2920.8 | -1462.8 (-1537.8 to -1387 | -50.1 | 728 | 1362.6 | -634.6 (-684 to -585.2) | -46.6 | 715 | 1540.2 | -825.2 (-881.5 to -768.9) | -53.6 |
| Oct | 1870 | 3112.2 | -1242.2 (-1311.3 to -1173 | -39.9 | 1070 | 1530.4 | -460.4 (-502.5 to -418.3) | -30.1 | 884 | 1678.0 | -794 (-849.2 to -738.8) | -47.3 |
| Nov | 1804 | 3049.0 | -1245 (-1314.2 to -1175.8 | -40.8 | 1043 | 1519.4 | -476.4 (-519.2 to -433.6) | -31.4 | 819 | 1683.8 | -864.8 (-922.4 to -807.2) | -51.4 |
| Dec | 1531 | 2480.6 | -949.6 (-1010 to -889.2) | -38.3 | 766 | 1209.2 | -443.2 (-484.5 to -401.9) | -36.7 | 630 | 1307.2 | -677.2 (-728.2 to -626.2) | -51.8 |
| Jan, 2021 | 955 | 2995.0 | -2040.0 (-2128.5 to -1951.5) | -68.1 | 336 | 1453.2 | -1117.2 (-1182.7 to -1051 | -76.9 | 349 | 1623.6 | -1274.6 (-1344.6 to -1204.0) | -78.5 |
| Feb | 1122 | 2973.6 | -1851.6 (-1935.9 to -1767.0) | -62.3 | 411 | 1437.4 | -1026.4(-1089.2 to -963.0) | -71.4 | 415 | 1688.8 | -1273.8 (-1343.8 to -1203.0) | -75.4 |
| Mar | 1637 | 3244.4 | -1607.4 (-1686.0 to -1528.8) | -49.5 | 736 | 1478.2 | -742.2 (-795.6 to -688.8) | -50.2 | 727 | 1785.2 | -1058.2 (-1122.0 to -994.4) | -59.3 |

### Figure S2. Pre-pandemic median deprivation quintile by quarter


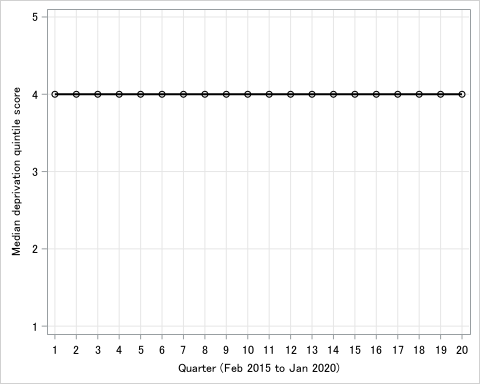


### Table S3: The difference between monthly elective dental admissions during the pandemic and the five-year pre-pandemic monthly mean by socioeconomic deprivation quintile for the under-11s

|  | 1 - Least deprived | | | | 2 | | | | 3 | | | | 4 | | | | 5 -Most deprived | | | |
| --- | --- | --- | --- | --- | --- | --- | --- | --- | --- | --- | --- | --- | --- | --- | --- | --- | --- | --- | --- | --- |
|  | O | E | Diff (95% CI) | % diff | O | E | Diff (95% CI) | % diff | O | E | Diff (95% CI) | % diff | O | E | Diff (95% CI) | % diff | O | E | Diff (95% CI) | % diff |
| Feb, 2020 | 280 | 290.8 | -10.8 (-17.2 to -4.4) | -3.7 | 340 | 366.4 | -26.4 (-36.5 to -16.3) | 7.2 | 441 | 473.0 | -32.2 (-43.3 to -21.1) | 6.8 | 621 | 696.4 | -75.4 (-92.4 to -58.4) | 3.7 | 1021 | 1139.0 | -118.0 (-139.3 to -96.7) | 10.4 |
| Mar | 179 | 309.4 | -130.4 (-152.8 to -108.0) | -42.1 | 207 | 393.4 | -186.4 (-213.2 to -159.6) | 47.4 | 270 | 518.4 | -248.4 (-279.3 to -217.5) | 47.9 | 357 | 754.0 | -397.0 (-436.1 to -357.9) | 42.1 | 675 | 1260.8 | -585.8 (-633.2 to -538.4) | 46.5 |
| Apr | 8 | 282.6 | -274.6 (-307.1 to -242.1) | -97.2 | 10 | 356.8 | -346.6 (-383.1 to -310.1) | 97.2 | 18 | 452.2 | -434.2 (-475 to -393.4) | 96.0 | 41 | 678.4 | -637.4 (-686.9 to -587.9) | 94.0 | 55 | 1128.4 | -1073.4(-1137.6 to -1009.0) | 95.1 |
| May | 25 | 297.0 | -272.0 (-304.3 to -239.7) | -91.6 | 43 | 385.0 | -342 (-378.2 to -305.8) | 88.8 | 48 | 496.0 | -448 (-489.5 to -406.5) | 90.3 | 66 | 734.4 | -668.4 (-719.1 to -617.7) | 91.6 | 100 | 1194.0 | -1094.0 (-1158.8 to -1029.2) | 91.6 |
| Jun | 48 | 298.0 | -250.0 (-281.0 to -219.0) | -83.9 | 55 | 367.8 | -312.8 (-347.5 to -278.1) | 85.0 | 57 | 488.6 | -431.6 (-472.3 to -390.9) | 88.3 | 95 | 735.2 | -640.2 (-689.8 to -590.6) | 83.9 | 195 | 1221.2 | -1026.2 (-1089 to -963.4) | 84.0 |
| Jul | 104 | 297.6 | -193.6 (-220.9 to -166.3) | -65.1 | 126 | 387.0 | -261 (-292.7 to -229.3) | 67.4 | 165 | 482.6 | -317.6 (-352.5 to -282.7) | 65.8 | 238 | 741.2 | -503.2 (-547.2 to -459.2) | 65.1 | 380 | 1198.6 | -818.6 (-874.7 to -762.5) | 68.3 |
| Aug | 124 | 296.0 | -172.0 (-197.7 to -146.3) | -58.1 | 138 | 392.2 | -254.2 (-285.4 to -223.0) | 64.8 | 199 | 476.0 | -277 (-309.6 to -244.4) | 58.2 | 262 | 700.8 | -438.8 (-479.9 to -397.7) | 58.1 | 418 | 1140.4 | -722.4 (-775.1 to -669.7) | 63.3 |
| Sep | 168 | 302.0 | -134.0 (-156.7 to -111.3) | -44.4 | 191 | 350.8 | -159.8 (-184.6 to -135.0) | 45.6 | 221 | 470.0 | -249 (-279.9 to -218.1) | 53.0 | 310 | 671.4 | -361.4 (-398.7 to -324.1) | 44.4 | 563 | 1089.6 | -526.6 (-571.6 to -481.6) | 48.3 |
| Oct | 217 | 308.4 | -91.4 (-110.1 to -72.7) | -29.6 | 275 | 390.6 | -115.6 (-136.7 to -94.7) | 29.6 | 298 | 488.8 | -190.8 (-217.9 to -163.7) | 39.0 | 446 | 711.4 | -265.4 (-297.3 to -233.5) | 29.6 | 627 | 1187.4 | -560.4 (-606.8 to -514).0 | 47.2 |
| Nov | 211 | 304.0 | -93.0 (-111.9 to -74.1) | -30.6 | 254 | 379.2 | -125.2 (-147.1 to -103.3) | 33.0 | 273 | 486.8 | -213.8 (-242.5 to -185.1) | 43.9 | 432 | 717.6 | -285.6 (-318.7 to -252.5) | 30.6 | 625 | 1122.6 | -497.6 (-541.3 to -453.9) | 44.3 |
| Dec | 165 | 243.4 | -78.4 (-95.8 to -61.0) | -32.2 | 213 | 291.4 | -78.4 (-95.8 to -61.0) | 26.9 | 236 | 393.4 | -157.4 (-182 to -132.8) | 40.0 | 348 | 573.8 | -225.8 (-255.3 to -196.3) | 32.2 | 555 | 957.8 | -402.8 (-442.1 to -363.5) | 42.1 |
| Jan, 2021 | 105 | 315.4 | -210.4 (-238.8 to -182.0) | -66.7 | 133 | 378.8 | -245.8 (-276.5 to -215.1) | 64.9 | 158 | 484.4 | -326.4 (-361.8 to -291.0) | 67.4 | 223 | 688.6 | -465.6 (-507.9 to -423.3) | 66.7 | 330 | 1118.2 | -788.2 (-843.2 to -733.2) | 70.5 |
| Feb | 124 | 290.8 | -166.8 (-192.1 to -141.4) | -57.4 | 159 | 366.4 | -207.4 (-235.6 to -179.2) | 56.6 | 163 | 473.2 | -310.2 (-344.7 to -275.7) | 65.6 | 264 | 696.4 | -426.5 (-467 to -386.0) | 57.4 | 409 | 1139.0 | -730.0 (-783.0 to -677.0) | 64.1 |
| Mar | 180 | 309.4 | -129.4 (-151.7 to -107.1) | -41.8 | 203 | 393.4 | -190.4 (-217.4 to -163.4) | 48.4 | 268 | 518.4 | -250.4 (-281.4 to -219.4) | 48.3 | 377 | 745.0 | -368.3 (-405.9 to -330.6) | 41.8 | 601 | 1260.8 | -659.8 (-710.1 to -609.5) | 52.3 |

### Table S4: The difference between monthly elective dental admissions during the pandemic and the five-year pre-pandemic monthly mean by socioeconomic deprivation quintile for the 11-16s

|  | 1 - Least deprived | | | | 2 | | | | 3 | | | | 4 | | | | 5 -Most deprived | | | |
| --- | --- | --- | --- | --- | --- | --- | --- | --- | --- | --- | --- | --- | --- | --- | --- | --- | --- | --- | --- | --- |
|  | O | E | Diff (95% CI) | % diff | O | E | Diff (95% CI) | % diff | O | E | Diff (95% CI) | % diff | O | E | Diff (95% CI) | % diff | O | E | Diff (95% CI) | % diff |
| Feb, 2020 | 265 | 268.4 | -3.4 (-7.0 to 0.2) | -1.3 | 238 | 264.4 | -24.5 (-34.2 to -14.8) | 10.0 | 250 | 268.2 | -18.2 (-26.6 to -9.8) | 6.8 | 250 | 283.0 | -33.0 (-44.3 to -21.7) | 11.7 | 341 | 351.0 | -10.0 (-16.2 to -3.8) | 2.8 |
| Mar | 150 | 286.6 | -136.6 (-159.5 to -113.7) | -47.7 | 147 | 275.2 | -121.3 (-142.8 to -99.7) | 46.6 | 149 | 263.0 | -114.0 (-134.9 to -93.1) | 43.3 | 146 | 287.2 | -141.2 (-164.5 to -117.9) | 49.2 | 205 | 363.0 | -158.0 (-182.6 to -133.4) | 43.5 |
| Apr | Supp | 274.0 | Supp | >98% | Supp | 246.4 | Supp (-275 to -213.8) | >98% | Supp | 248.6 | Supp(-278.4 to -216.8) | >98% | Supp | 258.0 | Supp (-285.2 to -222.8) | >98% | 5 | 318.6 | -313.6 (-348.3 to -278.9) | 98.4 |
| May | Supp | 258.8 | Supp | >98% | 5 | 241.2 | -236.2 (-266.3 to -206.1) | 97.9 | 5 | 239.0 | -234 (-264 to -204) | 97.9 | 7 | 261.2 | -254.2 (-285.4 to -223) | 97.3 | 9 | 324.6 | -315.6 (-350.4 to -280.8) | 97.2 |
| Jun | 12 | 254.0 | -242 (-272.5 to -211.5) | -95.3 | 19 | 256.6 | -237.6 (-267.8 to -207.4) | 92.6 | 19 | 264.4 | -245.4 (-276.1 to -214.7) | 92.8 | 17 | 270.2 | -253.2 (-284.4 to -222) | 93.7 | 17 | 343.4 | -326.4 (-361.8 to -291) | 95.0 |
| Jul | 47 | 268.4 | -221.4 (-250.6 to -192.2) | -82.5 | 48 | 268.0 | -220 (-249.1 to -190.9) | 82.1 | 46 | 260.0 | -214 (-242.7 to -185.3) | 82.3 | 49 | 291.0 | -242 (-272.5 to -211.5) | 83.2 | 72 | 357.8 | -285.8 (-318.9 to -252.7) | 79.9 |
| Aug | 97 | 275.6 | -178.6 (-204.8 to -152.4) | -64.8 | 115 | 258.2 | -143.2 (-166.7 to -119.7) | 55.5 | 100 | 244.4 | -144.4 (-168 to -120.8) | 59.1 | 101 | 289.8 | -188.8 (-215.7 to -161.9) | 65.1 | 94 | 323.2 | -229.2 (-258.9 to -199.5) | 70.9 |
| Sep | 148 | 267.6 | -119.6 (-141.0 to -98.2) | -44.7 | 144 | 269.0 | -125 (-146.9 to -103.1) | 46.5 | 129 | 233.8 | -104.8 (-124.9 to -84.7) | 44.8 | 129 | 271.4 | -142.4 (-165.8 to -119) | 52.5 | 176 | 313.6 | -137.6 (-160.6 to -114.6) | 43.9 |
| Oct | 234 | 299.6 | -65.6 (-81.5 to -49.7) | -21.9 | 204 | 283.0 | -79 (-96.4 to -61.6) | 27.9 | 167 | 286.4 | -119.4 (-140.8 to -98) | 41.7 | 210 | 292.8 | -82.8 (-100.6 to -65) | 28.3 | 254 | 362.6 | -108.6 (-129 to -88.2) | 30.0 |
| Nov | 197 | 299.2 | -102.2 (-122 to -82.4) | -34.2 | 215 | 277.8 | -62.8 (-78.3 to -47.3) | 22.6 | 187 | 279.4 | -92.4 (-111.2 to -73.6) | 33.1 | 189 | 283.8 | -94.8 (-113.9 to -75.7) | 33.4 | 251 | 371.8 | -120.8 (-142.3 to -99.3) | 32.5 |
| Dec | 151 | 224.6 | -73.6 (-90.4 to -56.8) | -32.8 | 149 | 217.8 | -68.8 (-85.1 to -52.5) | 31.6 | 149 | 219.2 | -70.2 (-86.6 to -53.8) | 32.0 | 143 | 245.4 | -102.4 (-122.2 to -82.6) | 41.7 | 171 | 298.6 | -127.6 (-149.7 to -105.5) | 42.7 |
| Jan, 2021 | 75 | 286.2 | -211.2 (-239.7 to -182.7) | -73.8 | 57 | 270.8 | -213.8 (-242.5 to -185.1) | 79.0 | 68 | 262.6 | -194.6 (-221.9 to -167.3) | 74.1 | 62 | 292.0 | -230.0 (-259.7 to -200.3) | 78.8 | 74 | 339.2 | -265.2 (-297.1 to -233.3) | 78.2 |
| Feb | 74 | 268.4 | -194.4 (-221.7 to -167.1) | -72.4 | 63 | 264.4 | -201.4 (-229.2 to -173.6) | 76.2 | 85 | 268.2 | -183.2 (-209.7 to -156.7) | 68.3 | 88 | 283.0 | -195.0 (-222.4 to -167.6) | 68.9 | 99 | 351.0 | -252.0 (-283.1 to -220.9) | 71.8 |
| Mar | 144 | 286.6 | -142.6 (-166.0 to -119.2) | -49.8 | 131 | 275.2 | -144.2 (-167.7 to -120.7) | 52.4 | 131 | 263.0 | -132.0 (-154.5 to -109.5) | 50.2 | 145 | 287.2 | -142.2 (-165.6 to -118.8) | 49.5 | 183 | 363.0 | -180.0 (-206.3 to -153.7) | 49.6 |

Supp: Suppressed due to small numbers

### Table S5: The difference between monthly elective dental admissions during the pandemic and the five-year pre-pandemic monthly mean by socioeconomic deprivation quintile for the 17-24s

|  | 1 - Least deprived | | | | 2 | | | | 3 | | | | 4 | | | | 5 -Most deprived | | | |
| --- | --- | --- | --- | --- | --- | --- | --- | --- | --- | --- | --- | --- | --- | --- | --- | --- | --- | --- | --- | --- |
|  | O | E | Diff (95% CI) | % diff | O | E | Diff (95% CI) | % diff | O | E | Diff (95% CI) | % diff | O | E | Diff (95% CI) | % diff | O | E | Diff (95% CI) | % diff |
| Feb, 2020 | 220 | 261 | -41.0 (-53.6 to -28.4) | -15.7 | 254 | 291.2 | -37.2 (-49.2 to -25.2) | 12.8 | 249 | 303.8 | -54.8 (-69.3 to -40.3) | 18.0 | 300 | 379 | -79.0 (-96.4 to -61.6) | 20.8 | 392 | 450.8 | -36.8 (-48.6 to -24.9) | 13.0 |
| Mar | 116 | 290.8 | -174.8 (-200.7 to -148.9) | -60.1 | 121 | 288.8 | -167.8 (-193.2 to -142.4) | 58.1 | 142 | 323.4 | -181.4(-207.8 to -155.0) | 56.1 | 149 | 388 | -239.0 (-269.3 to -208.7) | 61.6 | 222 | 486.8 | -241.8 (-272.2 to -211.3) | 54.4 |
| Apr | Supp | 242.8 | -241.8 (-272.3 to -211.3) | 98+ | 5 | 283.4 | -278.4 (-311.1 to -245.7) | 98.2 | 5 | 308.6 | -303.6 (-337.8 to -269.4) | 98.4 | 7 | 355 | -348 (-384.6 to -311.4) | 98%+ | 9 | 424.2 | -415.2 (-455.1 to -375.3) | 97.9 |
| May | 5 | 255.8 | -250.8 (-281.8 to -219.8) | -98.0 | 9 | 277.8 | -268.8 (-300.9 to -236.7) | 96.8 | 12 | 309.2 | -297.2 (-331 to -263.4) | 96.1 | 15 | 358 | -343 (-379.3 to -306.7) | 95.8 | 27 | 458.4 | -431.4 (-472.1 to -390.7) | 94.1 |
| Jun | 19 | 277 | -258 (-289.5 to -226.5) | -93.1 | 35 | 281 | -246 (-276.7 to -215.3) | 87.5 | 16 | 324 | -308 (-342.4 to -273.6) | 95.1 | 27 | 362 | -335 (-370.9 to -299.1) | 92.5 | 37 | 473.6 | -436.6 (-477.6 to -395.6) | 92.2 |
| Jul | 62 | 272 | -210 (-238.4 to -181.6) | -77.2 | 65 | 285.6 | -220.6 (-249.7 to -191.5) | 77.2 | 52 | 321.6 | -269.6 (-301.8 to -237.4) | 83.8 | 82 | 353.6 | -271.6 (-303.9 to -239.3) | 76.8 | 93 | 448.4 | -355.4 (-392.4 to -318.4) | 79.3 |
| Aug | 91 | 266 | -175 (-200.9 to -149.1) | -65.8 | 91 | 267.4 | -176.4 (-202.4 to -150.4) | 66.0 | 90 | 316.2 | -226.2 (-255.7 to -196.7) | 71.5 | 104 | 363 | -259 (-290.5 to -227.5) | 71.3 | 123 | 431.2 | -308.2 (-342.6 to -273.8) | 71.5 |
| Sep | 127 | 241 | -114 (-134.9 to -93.1) | -47.3 | 112 | 268.8 | -156.8 (-181.3 to -132.3) | 58.3 | 121 | 287.6 | -166.6 (-191.9 to -141.3) | 57.9 | 149 | 335.2 | -186.2 (-212.9 to -159.5) | 55.5 | 206 | 400.2 | -194.2 (-221.5 to -166.9) | 48.5 |
| Oct | 146 | 256.6 | -110.6 (-131.2 to -90.0) | -43.1 | 159 | 299 | -140 (-163.2 to -116.8) | 46.8 | 160 | 303.2 | -143.2 (-166.7 to -119.7) | 47.2 | 187 | 362 | -175 (-200.9 to -149.1) | 48.3 | 228 | 448.8 | -220.8 (-249.9 to -191.7) | 49.2 |
| Nov | 128 | 250 | -122 (-143.6 to -100.4) | -48.8 | 147 | 285.4 | -138.4 (-161.5 to -115.3) | 48.5 | 158 | 323.4 | -165.4 (-190.6 to -140.2) | 51.1 | 181 | 353.4 | -172.4 (-198.1 to -146.7) | 48.8 | 204 | 461.8 | -257.8 (-289.3 to -226.3) | 55.8 |
| Dec | 112 | 210.8 | -98.8 (-118.3 to -79.3) | -46.9 | 97 | 219.6 | -122.6 (-144.3 to -100.9) | 55.8 | 120 | 232.6 | -112.6 (-133.4 to -91.8) | 48.4 | 144 | 289 | -145 (-168.6 to -121.4) | 50.2 | 155 | 351.4 | -196.4 (-223.9 to -168.9) | 55.9 |
| Jan, 2021 | 47 | 262.4 | -215.4 (-244.2 to -186.6) | -82.1 | 58 | 270.8 | -212.8 (-241.4 to -184.2) | 78.6 | 64 | 300.8 | -236.8 (-267.0 to -206.6) | 78.7 | 87 | 351.8 | -264.8 (-296.7 to -232.9) | 75.3 | 90 | 432.8 | -342.8 (-379.1 to -306.5) | 79.2 |
| Feb | 62 | 261 | -199.0 (-226.6 to -174.4) | -76.2 | 70 | 291.2 | -221.2 (-250.4 to -192.0) | 76.2 | 73 | 303.8 | -230.8 (-260.6 to -201.0) | 76.0 | 87 | 379 | -292.0 (-325.5 to -258.5) | 77.0 | 121 | 450.8 | -329.8 (-365.4 to -294.2) | 73.2 |
| Mar | 110 | 290.8 | -180.8 (-207.2 to -154.4) | -62.2 | 113 | 288.8 | -175.8 (-201.8 to -149.8) | 60.9 | 140 | 323.4 | -183.4 (-209.9 to -156.9) | 56.7 | 145 | 388 | -243.0 (-273.6 to -212.4) | 62.6 | 218 | 486.8 | -268.8 (-300.9 to -236.7) | 55.2 |

Supp: Suppressed due to small numbers

### Table S6: Standard residuals for the chi-squared test between pandemic periods by socioeconomic deprivation quintiles for the under-11s

| **Period** | **Quintile** | **Observed** | **Expected** | **Standardised residuals** |
| --- | --- | --- | --- | --- |
| Pre-COVID | 1 -Least deprived | 17723 | 17891.2096 | -1.2575665 |
| Pre-COVID | 2 | 22197 | 22334.665 | -0.9211574 |
| Pre-COVID | 3 | 28552 | 28543.4908 | 0.05036595 |
| Pre-COVID | 4 | 42016 | 41946.6557 | 0.33858048 |
| Pre-COVID | 5 – Most deprived | 68789 | 68560.9789 | 0.87083637 |
| COVID | 1 -Least deprived | 1938 | 1769.79039 | 3.99843366 |
| COVID | **2** | 2347 | 2209.33499 | 2.92882067 |
| COVID | 3 | 2815 | 2823.50924 | -0.1601386 |
| COVID | 4 | 4080 | 4149.34427 | -1.0765169 |
| COVID | 5 – Most deprived | 6554 | 6782.02112 | -2.7688249 |

### Table S7: Standard residuals for the chi-squared test between pandemic periods by ethnicity for the under-11s

| **Period** | **Ethnic group** | **Observed** | **Expected** | **Standardised residuals** |
| --- | --- | --- | --- | --- |
| 0 | White | 107876 | 107385.188 | 1.49776338 |
| 0 | Other inc mixed | 13021 | 13065.8714 | -0.3925552 |
| 0 | Asian/asian british | 16770 | 16655.0264 | 0.89089246 |
| 0 | Black/black british | 5334 | 5318.64893 | 0.21049311 |
| 0 | Unknown | 37925 | 38501.2654 | -2.9368723 |
| 1 | White | 10096 | 10586.8123 | -4.7701583 |
| 1 | Other inc mixed | 1333 | 1288.12857 | 1.25023125 |
| 1 | Asian/asian british | 1527 | 1641.97355 | -2.8373628 |
| 1 | Black/black british | 509 | 524.351066 | -0.6703899 |
| 1 | Unknown | 4372 | 3795.73456 | 9.35351086 |

### Table S8: Standard residuals for the chi-squared test between pandemic periods by ethnicity for the 11-16s

| **Period** | **Ethnic group** | **Observed** | **Expected** | **Standardised residuals** |
| --- | --- | --- | --- | --- |
| 0 | White | 55989 | 55836.5825 | 0.64502337 |
| 0 | Other inc mixed | 3958 | 4001.94559 | -0.6946718 |
| 0 | Asian/asian british | 6251 | 6208.62586 | 0.53777812 |
| 0 | Black/black british | 2811 | 2791.41862 | 0.37062164 |
| 0 | Unknown | 15602 | 15772.4274 | -1.3570325 |
| 1 | White | 5220 | 5372.41751 | -2.0794572 |
| 1 | Other inc mixed | 429 | 385.054414 | 2.2395162 |
| 1 | Asian/asian british | 555 | 597.374137 | -1.7337148 |
| 1 | Black/black british | 249 | 268.581378 | -1.1948278 |
| 1 | Unknown | 1688 | 1517.57256 | 4.37486635 |

### Table S9: Standard residuals for the chi-squared test between pandemic periods by ethnicity for the 17-24s

| **Period** | **Ethnic group** | **Observed** | **Expected** | **Standardised residuals** |
| --- | --- | --- | --- | --- |
| 0 | White | 64217 | 63901.4202 | 1.24840033 |
| 0 | Other inc mixed | 4183 | 4213.83026 | -0.4749399 |
| 0 | Asian/asian british | 4243 | 4312.04178 | -1.0514062 |
| 0 | Black/black british | 3822 | 3884.91431 | -1.0093894 |
| 0 | Unknown | 21854 | 22006.7934 | -1.0299739 |
| 1 | White | 4752 | 5067.57975 | -4.4331133 |
| 1 | Other inc mixed | 365 | 334.169739 | 1.68652835 |
| 1 | Asian/asian british | 411 | 341.958216 | 3.73358022 |
| 1 | Black/black british | 371 | 308.085689 | 3.58437699 |
| 1 | Unknown | 1898 | 1745.2066 | 3.65747326 |

### Table S10: Count ratios of admissions after modelling the interaction between socioeconomic deprivation quintile and ethnicity by pandemic period and age group.

|  |  | 0-10 years | | 11-16 years | | 17-24 years | |
| --- | --- | --- | --- | --- | --- | --- | --- |
|  | Deprivation quintile | Count ratio^1^ (95% CI) | P-value | Count ratio^1^ (95% CI) | P-value | Count ratio^1^ (95% CI) | P-value |
| Intercept |  | 1216.96 (1216.44 to 1217. | <.0001 | 1148.92 (1148.44 to 1149. | <.0001 | 850.1 (849.69 to 850.51) | <.0001 |
| Pandemic period | Pre-pandemic | 10.47 (10.46 to 10.47) | <.0001 | 10.55 (10.55 to 10.55) | <.0001 | 13.14 (13.13 to 13.15) | <.0001 |
|  | Pandemic | 1 (1 to 1) |  | 1 (1 to 1) |  | 1 (1 to 1) |  |
| White | Second least depr. | 1.23 (1.23 to 1.23) | <.0001 | 0.96 (0.96 to 0.96) | <.0001 | 1.11 (1.11 to 1.11) | <.0001 |
| White |  | 1.5 (1.5 to 1.5) | <.0001 | 0.9 (0.9 to 0.9) | <.0001 | 1.17 (1.16 to 1.16) | <.0001 |
| White |  | 2.08 (2.08 to 2.08) | <.0001 | 0.9 (0.9 to 0.9) | <.0001 | 1.26 (1.26 to 1.26) | <.0001 |
| White | Most depr.  Relative diff | 2.62 (2.62 to 2.62) | <.0001 | 0.84 (0.84 to 0.84) | <.0001 | 1.2 (1.2 to 1.2) | <.0001 |
| White | Unknown | 0.05 (0.04 to 0.04) | <.0001 | 0.01 (0.01 to 0.01) | <.0001 | 0.02 (0.02 to 0.02) | <.0001 |
| Other^2^ | Least depr. | 0.06 (0.06 to 0.06) | <.0001 | 0.04 (0.04 to 0.04) | <.0001 | 0.03 (0.03 to 0.03) | <.0001 |
| Other^2^ |  | 0.08 (0.08 to 0.08) | <.0001 | 0.04 (0.04 to 0.04) | <.0001 | 0.04 (0.03 to 0.03) | <.0001 |
| Other^2^ |  | 0.13 (0.13 to 0.13) | <.0001 | 0.05 (0.05 to 0.05) | <.0001 | 0.05 (0.05 to 0.05) | <.0001 |
| Other^2^ |  | 0.22 (0.22 to 0.22) | <.0001 | 0.07 (0.07 to 0.07) | <.0001 | 0.09 (0.08 to 0.08) | <.0001 |
| Other^2^ | Most depr.  0.52/0.06 = relative diff ~8 | 0.52 (0.52 to 0.52) | <.0001 | 0.13 (0.12 to 0.13) | <.0001 | 0.17 (0.17 to 0.17) | <.0001 |
| Other^2^ | Unknown | 0.01 (0.01 to 0.01) | <.0001 | 0 (0 to 0) | <.0001 | 0 (0 to 0) | <.0001 |
| Asian/Asian British | Least depr. | 0.04 (0.04 to 0.04) | <.0001 | 0.04 (0.04 to 0.04) | <.0001 | 0.02 (0.02 to 0.02) | <.0001 |
| Asian/Asian British |  | 0.07 (0.07 to 0.07) | <.0001 | 0.04 (0.04 to 0.04) | <.0001 | 0.03 (0.03 to 0.03) | <.0001 |
| Asian/Asian British |  | 0.13 (0.13 to 0.13) | <.0001 | 0.07 (0.06 to 0.07) | <.0001 | 0.05 (0.05 to 0.05) | <.0001 |
| Asian/Asian British |  | 0.24 (0.24 to 0.24) | <.0001 | 0.11 (0.11 to 0.11) | <.0001 | 0.08 (0.08 to 0.08) | <.0001 |
| Asian/Asian British | Most depr.  0.81/0.04 = 20 fold difference | 0.81 (0.81 to 0.81) | <.0001 | 0.26 (0.26 to 0.26) | <.0001 | 0.21 (0.2 to 0.21) | <.0001 |
| Asian/Asian British | Unknown | 0.03 (0.03 to 0.03) | <.0001 | 0.01 (0 to 0.01) | <.0001 | 0 (0 to 0) | <.0001 |
| Black/Black British | Least depr. | 0.01 (0.01 to 0.01) | <.0001 | 0.01 (0.01 to 0.01) | <.0001 | 0.01 (0.01 to 0.01) | <.0001 |
| Black/Black British |  | 0.02 (0.01 to 0.02) | <.0001 | 0.01 (0.01 to 0.01) | <.0001 | 0.01 (0.01 to 0.01) | <.0001 |
| Black/Black British |  | 0.03 (0.03 to 0.03) | <.0001 | 0.02 (0.02 to 0.02) | <.0001 | 0.03 (0.03 to 0.03) | <.0001 |
| Black/Black British |  | 0.08 (0.08 to 0.08) | <.0001 | 0.05 (0.05 to 0.05) | <.0001 | 0.07 (0.07 to 0.07) | <.0001 |
| Black/Black British | Most depr. | 0.28 (0.28 to 0.28) | <.0001 | 0.14 (0.14 to 0.14) | <.0001 | 0.21 (0.21 to 0.21) | <.0001 |
| Black/Black British | Unknown | 0.01 (0.01 to 0.01) | <.0001 | 0 (0 to 0) | <.0001 | 0 (0 to 0) | <.0001 |
| Unknown | Least depr. | 0.28 (0.28 to 0.28) | <.0001 | 0.26 (0.26 to 0.26) | <.0001 | 0.32 (0.32 to 0.32) | <.0001 |
| Unknown |  | 0.34 (0.34 to 0.34) | <.0001 | 0.23 (0.23 to 0.23) | <.0001 | 0.3 (0.3 to 0.3) | <.0001 |
| Unknown |  | 0.45 (0.45 to 0.45) | <.0001 | 0.23 (0.23 to 0.23) | <.0001 | 0.34 (0.34 to 0.34) | <.0001 |
| Unknown |  | 0.69 (0.69 to 0.69) | <.0001 | 0.24 (0.24 to 0.24) | <.0001 | 0.41 (0.41 to 0.41) | <.0001 |
| Unknown | Most depr. | 1.18 (1.18 to 1.18) | <.0001 | 0.32 (0.32 to 0.32) | <.0001 | 0.58 (0.57 to 0.57) | <.0001 |
| Unknown | Unknown | 0.04 (0.04 to 0.04) | <.0001 | 0.01 (0.01 to 0.01) | <.0001 | 0.01 (0.01 to 0.01) | <.0001 |
| Ref (White, Least depr) | White, least depr. | 1 (1 to 1) |  | 1 (1 to 1) |  | 1 (1 to 1) |  |

^1^Ratio of the counts of admissions; ^2^Other including mixed
